# Bayesian Estimation of Nonsynonymous/Synonymous Rate Ratios for Pairwise Sequence Comparisons

**DOI:** 10.1093/molbev/msu142

**Published:** 2014-04-18

**Authors:** Konstantinos Angelis, Mario dos Reis, Ziheng Yang

**Affiliations:** ^1^Department of Genetics, Evolution and Environment, University College London, London, United Kingdom

**Keywords:** nonsynonymous/synonymous rate ratio, evolutionary distance, Bayesian estimation, pairwise comparisons, protein-coding sequences

## Abstract

The nonsynonymous/synonymous rate ratio (*ω* = *d*_N_/*d*_S_) is an important measure of the mode and strength of natural selection acting on nonsynonymous mutations in protein-coding genes. The simplest such analysis is the estimation of the *d*_N_/*d*_S_ ratio using two sequences. Both heuristic counting methods and the maximum-likelihood (ML) method based on a codon substitution model are widely used for such analysis. However, these methods do not have nice statistical properties, as the estimates can be zero or infinity in some data sets, so that their means and variances are infinite. In large genome-scale comparisons, such extreme estimates (either 0 or ∞) of *ω* and sequence distance (*t*) are common. Here, we implement a Bayesian method to estimate *ω* and *t* in pairwise sequence comparisons. Using a combination of computer simulation and real data analysis, we show that the Bayesian estimates have better statistical properties than the ML estimates, because the prior on *ω* and *t* shrinks the posterior of those parameters away from extreme values. We also calculate the posterior probability for *ω* > 1 as a Bayesian alternative to the likelihood ratio test. The new method is computationally efficient and may be useful for genome-scale comparisons of protein-coding gene sequences.

## Introduction

The nonsynonymous/synonymous rate ratio (*ω* = *d*_N_/*d*_S_) is an important measure of the mode and strength of natural selection acting on protein-coding genes (Kimura 1977). A number of methods have been developed to estimate *ω* from pairwise sequence alignments, ranging from heuristic counting methods (Li et al. 1985; Nei and Gojobori 1986; Yang and Nielsen 2000) to maximum-likelihood (ML) methods based on an explicit Markov model of codon evolution (Goldman and Yang 1994). ML estimates (MLEs) of *ω* for thousands of genes are routinely calculated as descriptive statistics in genomic comparisons (Nielsen et al. 2005; Ge et al. 2008; Walters and Harrison 2010; Buschiazzo et al. 2012; Gladieux et al. 2013; Wang and Chen 2013). Although the ML method for pairwise comparisons produces reasonable estimates of *ω* and sequence distance (*t*) for most data sets, it suffers from a few problems when the data sets are extreme. For example, the MLE of *ω* (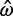) is 0 when the two compared sequences have only synonymous differences and ∞ when they have only nonsynonymous differences. Similarly, when the sequences are identical, the MLE 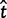 is 0 and 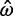 is not unique. When the sequences are very divergent 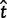 may be ∞.

Because of these infinite or undefined estimates, neither 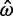 nor 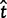 have finite means or variances. Extreme values of 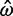 and 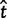 are commonly encountered in genome-level comparisons of thousands of genes, and those extreme estimates cause difficulties with the calculation of summary statistics (such as mean 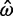 and 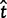 across all genes in the genome). An estimation method that always produces finite and reasonable estimates for *ω* and *t* is thus desirable. Here, we develop a Bayesian method to calculate the posterior means of *ω* and *t* between two sequences, denoted 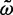 and 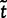. Using computer simulation, we show that the posterior means of *ω* and *t* are well behaved and have better Frequentist properties than the MLEs. We then use ML and the new Bayesian method to estimate *ω* and *t* from pairwise gene alignments for the genomes of four mammals (human, chimpanzee, mouse, and rat) and three bacterial strains (*Escherichia coli* O157:H7, *E. coli* K-12, and *Salmonella typhimurium* LT2). We show that extreme MLEs of *ω* and *t* are common in these data sets, and that the Bayesian method produces finite, well-behaved estimates. The new Bayesian method is computationally efficient and is implemented in the CODEML program of the PAML package (Yang 2007).

## New Bayesian Approach to Estimate *ω* and *t*

Here, we summarize the main features of the new Bayesian approach. The joint posterior distribution of *t* and *ω* given the data *x* (the pairwise sequence alignment) is
(1)
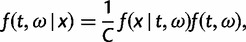

where *f*(*x|t*, *ω*) is the likelihood or the probability of observing the data *x* given *t* and *ω*, *f*(*t*, *ω*) is the prior and *C* = ∫∫ *f*(*x*|*t*, *ω*) *f*(*t*, *ω*)d*t*d*ω* is the normalizing constant. The posterior is proportional to the product of the likelihood and the prior. If the model involves the transition/transversion rate ratio (*κ*), its MLE (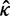) is used. If the model involves nucleotide or codon frequency parameters, they are estimated using the observed frequencies. When the data are informative, the likelihood dominates the posterior. When the data are uninformative, the prior may have a strong influence on the posterior. Here, we use two independent gamma distributions to construct the joint prior of *t* and *ω*:
(2)
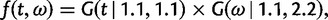

where the gamma density *G*(*x*|*α*, *β*) has mean *α*/*β* and variance *α*/*β*^2^. Here, the prior means of *t* and *ω* are 1 and 0.5, respectively, and the shape parameter *α* = 1.1 indicates that the priors are quite diffuse. This joint prior has a mode away from (0,0) and the prior density decays to 0 as either *t* or *ω* approaches ∞, thus penalizing extreme values. The likelihood is calculated from a pairwise sequence alignment using a codon substitution model (Yang and Nielsen 1998). As point estimates of *ω* and *t* we use their posterior means
(3)
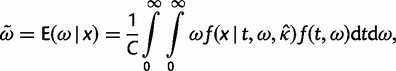

(4)
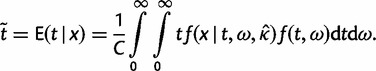



The posterior variances and covariance of *ω* and *t* can be similarly defined and can be calculated using standard numerical techniques. We use Gaussian quadrature to calculate all integrals numerically. We use similar techniques to calculate *P*(*ω* > 1|*x*), the posterior probability that *ω* > 1, which may be compared with the likelihood ratio test (LRT) of the null hypothesis *H*_0_: *ω* = 1 (see Methods and Materials).

**Fig. 1.**
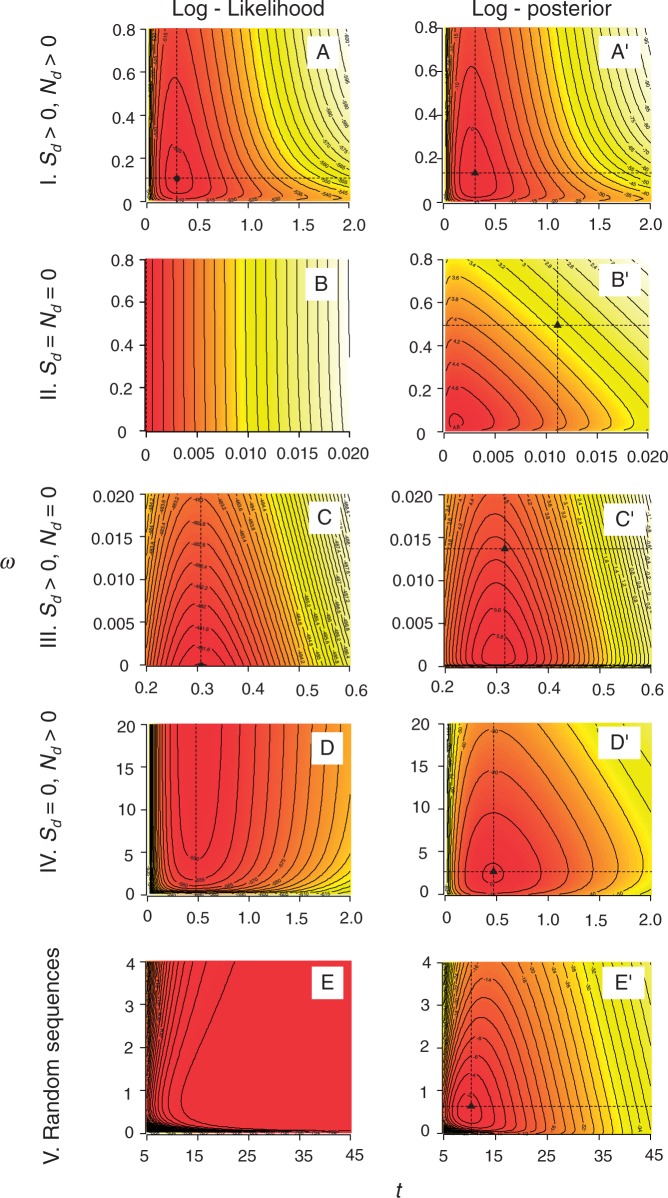
Contour plots of log-likelihood (A–E) and log-posterior (A'–E') densities for *ω* and *t* for five synthetic pairwise sequence alignments of 100 codons. The dashed lines indicate the MLE. Five cases are analyzed: I. normal sequences (A and A'), II. identical sequences (B and B'), III. sequences with only synonymous changes (C and C'), IV. with only nonsynonymous changes (D and D'), V. random sequences (E and E').

We consider five different scenarios in which the numerical calculations of the integrals may differ. We simulated five data sets to represent those five scenarios, each consisting of 2 sequences of 100 codons, with different numbers of synonymous (*S*_d_) and nonsynonymous (*N*_d_) differences. The posterior and likelihood surfaces for the five cases are shown in figure 1.

*Case I:* (*S*_d_ > 0, *N*_d_ > 0). This is the most common case, with both synonymous and nonsynonymous differences observed. The data are quite informative about *ω* and *t* and the posterior distribution resembles the likelihood (fig. 1*A*′ and *A*). In our example data set, we have *S* = 73.7, *N* = 226.3, *S*_d_ = 18.5, *N*_d_ = 6.5, where *S* and *N* are the numbers of synonymous and nonsynonymous sites. The MLEs are 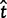 = 0.30 and 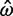 = 0.11 whereas the posterior means are 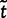 = 0.31 and 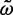 = 0.13.

*Case II:* (*S*_d_ = *N*_d_ = 0). In this case, the two sequences are identical. The likelihood is maximized when *t* = 0 and when *t* = 0, *ω* has no effect on the likelihood, so the MLE of *ω* is not unique (fig. 1*B*). In our example, *S* = 73.3, *N* = 226.7, *S*_d_ = *N*_d_ = 0. The posterior has a single mode and the posterior means are 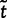 = 0.011 and 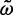 = 0.496 (fig. 1*B′*). Note that the posterior mean of *ω* is almost equal to the prior mean, since the data are uninformative about *ω*. Also, the posterior mean is markedly different from the posterior mode, because the posterior distribution is highly skewed.

*Case III:* (*S*_d_ > 0, *N*_d_ = 0). Only synonymous differences are observed. In our example, *S* = 74.4, *N* = 225.6, *S*_d_ = 24 and *N*_d_ = 0. Then, we have 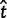 = 0.306 and 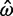 = 0 (fig. 1*C*). The posterior for *ω* has a mode away from 0 and 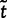 = 0.316 and 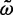 = 0.014 (fig. 1*C'*).

*Case IV:* (*S*_d_ >> 0, *N*_d_ >> 0). Only nonsynonymous differences are observed. In our example, *S* = 73.2, *N* = 226.8, *S*_d_ = 0, *N*_d_ = 40. The MLEs are 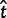 = 0.48 and 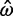 = ∞ (fig. 1*D*). The posterior has a well-defined mode and thus 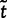 = 0.47 and 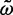 = 3.1 (fig. 1*D'*).

*Case V:* (*S*_d_ >> 0, *N*_d_ >> 0). The two sequences are so divergent that they look like random sequences (*S* = 75.9, *N* = 224.1, *S*_d_ = 75, *N*_d_ = 175). Here, the likelihood increases with the increase of both *t* and *ω,* with the MLEs at 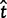 = ∞ and 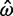 = ∞ (fig. 1*E*). In the Bayesian analysis, the prior penalizes large values and thus the posterior means are 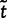 = 10.31 and 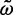 = 0.72 (fig. 1*E'*). Note that the posterior mean of *ω* is close to the prior mean, since the data of two nearly random sequences are uninformative about *ω*.

These five cases illustrate how the prior influences the posterior depending on whether the data are informative or not. The posterior means of *t* and *ω* are finite for all five cases, whereas the MLEs are not. We note that because the MLEs of *t* and *ω* may be infinite, their mean square errors (MSEs) are ∞ as well. The MSEs of the posterior means are in contrast always well defined. In this sense, the posterior mean estimates have better Frequentist properties than the MLEs. In the next section, we study the statistical properties of the Bayesian estimates of *t* and *ω* using simulated and real data, in comparison with the MLEs. We calculate the MSEs of the MLEs by excluding the infinite estimates.

## Results

### Analysis of Simulated Data

To examine the statistical properties of the posterior estimates of *t* and *ω*, we conducted a computer simulation. The program EVOLVER from the PAML package (Yang 2007) was used to generate pairwise sequence alignments of length *L*_c_ = 500 codons. We used *t* = 0.1, 0.5, 1, and 5 and *ω* = 0.01, 0.1, 0.5, and 2 (16 combinations) with transition/transversion rate ratio *κ* = 2 and equal codon frequencies (1/61) to generate the data sets. The number of replicates was 10,000. The simulated data sets were analyzed using both ML and the new Bayesian method using the CODEML program (Yang 2007). The same prior (eq. 2) was used for all data sets. Equal codon frequencies are assumed in the model (Fequal model).

**Fig. 2.**
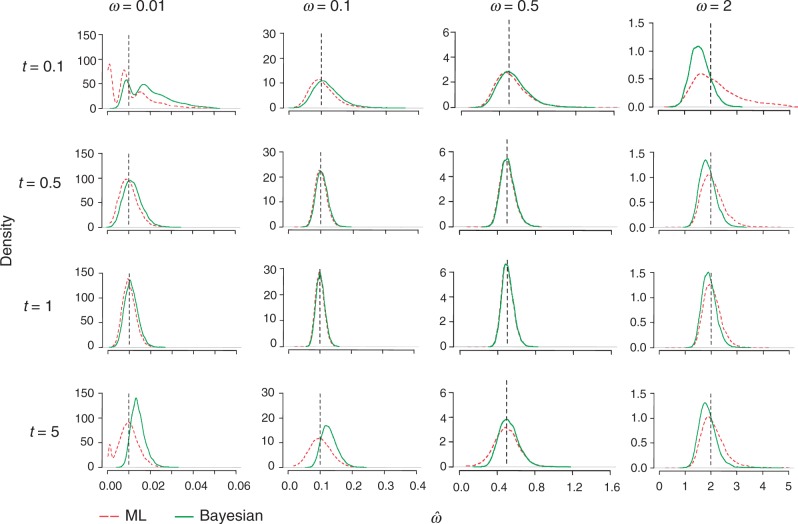
Kernel density (smoothed histogram) of MLEs (dashed red) and Bayesian posterior means (solid green) for *ω* in simulated data sets. The true values of *ω* and *t* are shown on the top and left of the plots, respectively. The sequence length is 500 codons. The number of replicates is 10,000. The vertical dashed lines correspond to the true values of *ω*. Independent gamma priors are used *ω ∼ G*(1.1, 2.2), *t ∼ G*(1.1, 1.1) (eq. 2).

**Fig. 3.**
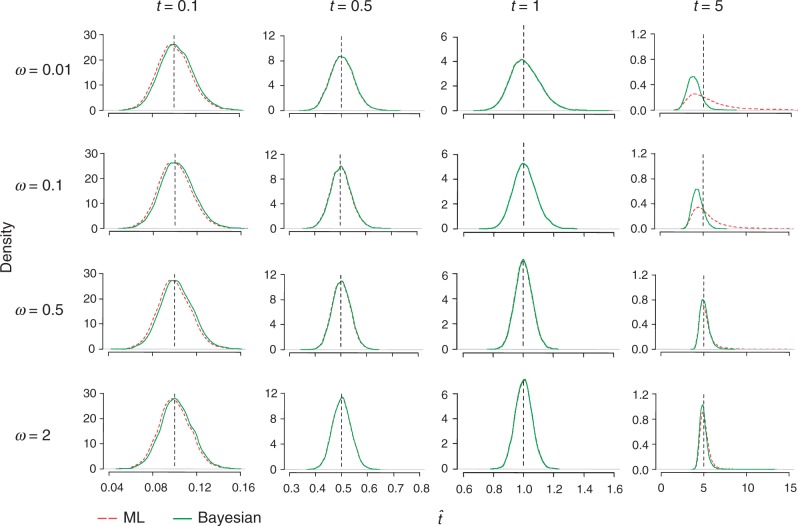
Kernel density (smoothed histogram) of MLEs (dashed red) and Bayesian posterior means (solid green) for *t* in simulated data sets. Details as in figure 2.

Figures 2 and 3 show the histograms (smoothed densities) of posterior mean estimates and MLEs of *t* and *ω*. As we see in figure 2, ML and Bayesian results are nearly identical for all combinations of *ω* = 0.1 and 0.5 and *t* = 0.5 and 1. However, for *ω* = 0.01, Bayesian estimates of *ω* are shifted to the right (too large) for all *t* values, as the prior for *ω* has a mean of 0.5 and affects the posterior estimates. For *ω* = 2, posterior estimates of *ω* are shifted to the left (too small) due to the prior. Generally, both methods behave best (estimates are more concentrated around the true value) for intermediate distances (*t* = 0.5 and 1), because sequences of moderate divergences are the most informative. The estimates of *t* show similar patterns (fig. 3). Although for *t* = 0.5 and 1 the Bayesian estimates are almost identical to the MLEs, for *t* = 0.1 Bayesian results are slightly shifted to the right (too large) and for *t* = 5 they are shifted to the left (too small).

The means of the Bayesian and ML estimates, the square root of the MSE (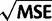), and the 2.5% and 97.5% percentiles of estimates from the 10,000 simulations are presented in tables 1 and 2. Those for the ML method are calculated after the infinite estimates are removed. We see that for highly similar (*t* = 0.1) and highly divergent (*t* = 5) sequences, the prior has a noticeable impact. For example, when *t* = 0.1 the mean of Bayesian estimates of *ω* is 0.02 when the true *ω* = 0.01 and is 1.591 when the true *ω* = 2.0. The mean MLEs are in comparison closer to the true values than the means of Bayesian estimates. However, the means for the MLEs are calculated after data sets in which 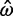 = ∞ are excluded, whereas those same data sets are included in the calculation of the Bayesian estimates. Similar patterns are observed concerning estimates of *t*. Moreover, for small and intermediate *ω* and *t*, ML and Bayesian methods have similar MSE, but for large *ω* and *t*, the Bayesian has smaller MSE indicating that in those cases Bayesian estimates are preferable to the MLEs.

**Table 1.** Summary Statistics of Bayesian (top, underlined) and ML (bottom) Estimates of 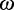 from 10,000 Simulated Data Sets.

	*ω* = 0.01					*ω* = 0.1				*ω* = 0.5					*ω* = 2					
	Mean	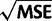	2.5%	97.5%	*N*_0_	Mean	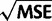	2.5%	97.5%	Mean	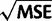	2.5%	97.5%	*N*_∞_	Mean	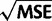	2.5%	97.5%	*N*_∞_	*P*_+_
*t* = 0.1	0.020	0.014	0.007	0.044	0	0.118	0.045	0.052	0.214	0.543	0.160	0.301	0.904	0	1.591	0.546	0.966	2.359	0	35.1
	0.011	0.009	0	0.033	2861	0.103	0.039	0.041	0.194	0.528	0.172	0.278	0.936	0	2.365	1.484	1.015	5.626	3	60.7
*t* = 0.5	0.012	0.005	0.005	0.021	0	0.104	0.018	0.072	0.141	0.511	0.076	0.379	0.677	0	1.878	0.329	1.360	2.543	0	98.3
	0.010	0.004	0.003	0.019	15	0.101	0.018	0.069	0.138	0.506	0.076	0.374	0.674	0	2.064	0.424	1.409	3.031	0	98.9
*t* = 1	0.011	0.003	0.006	0.018	0	0.102	0.014	0.076	0.132	0.506	0.062	0.397	0.637	0	1.922	0.278	1.466	2.497	0	99.9
	0.010	0.003	0.005	0.017	0	0.100	0.014	0.075	0.130	0.503	0.062	0.393	0.635	0	2.038	0.326	1.508	2.764	0	100
*t* = 5	0.014	0.005	0.009	0.022	0	0.129	0.038	0.089	0.183	0.526	0.109	0.348	0.755	0	1.876	0.374	1.331	2.642	0	97.4
	0.010	0.005	0	0.019	370	0.101	0.034	0.037	0.171	0.515	0.981	0.226	0.762	44	2.120	1.398	1.400	3.228	0	98.6

Note.—The Fequal model is used for codon frequencies. Results for ML have been calculated after removing infinite estimates. For *ω* = 0.1, there were no data sets with 0 or infinite estimates. *N*_0_ is the number of replicates with 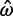 = 0, whereas *N*_∞_ is the number of replicates with 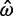 = ∞. *P*_+_ is the proportion of replicates with significant evidence for positive selection indicated by *P*(*ω* > 1 | *x*) > 0.95 in the Bayesian method or by a significant LRT at the 5% level (one-sided with critical value 2.71) in the likelihood method.

**Table 2.** Summary Statistics of Bayesian (top, underlined) and ML (bottom) Estimates of *t* from 10,000 Simulated Data Sets.

	*t* = 0.1				*t* = 0.5				*t* = 1				*t* = 5				
	Mean	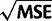	2.5%	97.5%	Mean	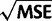	2.5%	97.5%	Mean	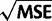	2.5%	97.5%	Mean	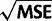	2.5%	97.5%	*N*_∞_
*ω* = 0.01	0.102	0.015	0.074	0.134	0.504	0.045	0.421	0.596	1.013	0.100	0.837	1.223	3.910	1.322	2.600	5.506	0
	0.100	0.015	0.072	0.132	0.503	0.045	0.419	0.595	1.011	0.100	0.836	1.222	7.572	8.922	2.676	43.744	244
*ω* = 0.1	0.102	0.015	0.075	0.133	0.503	0.041	0.427	0.587	1.007	0.077	0.865	1.171	4.406	0.869	3.317	5.795	0
	0.100	0.015	0.073	0.131	0.502	0.041	0.425	0.585	1.006	0.077	0.864	1.170	5.629	2.700	3.373	11.506	24
*ω* = 0.5	0.102	0.015	0.075	0.132	0.503	0.036	0.436	0.574	1.004	0.057	0.895	1.118	5.158	1.469	4.249	6.368	0
	0.100	0.015	0.073	0.130	0.501	0.036	0.434	0.572	1.002	0.057	0.894	1.116	5.440	2.601	4.228	7.979	43
*ω* = 2	0.102	0.015	0.075	0.131	0.501	0.035	0.434	0.571	1.001	0.056	0.895	1.112	4.988	0.737	4.274	6.035	0
	0.100	0.014	0.073	0.129	0.500	0.035	0.433	0.571	1.002	0.056	0.895	1.114	5.119	0.726	4.323	6.401	3

Note.—The Fequal model is used for codon frequencies. Results for ML have been calculated after removing the infinite estimates. For *t* = 0.1, 0.5, and 1, there were no data sets with 0 or infinite estimate. *N*_∞_ is the number of replicates with 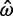 = ∞.

We also considered a test of positive selection, indicated by *ω* > 1. For ML, a LRT is used to compare H_0_: *ω* = 1 against H_1_: *ω* > 1, at the 5% significance level. In the Bayesian framework, we require the posterior probability to exceed the threshold *P*(*ω* > 1 | *x*) > 0.95. For the true *ω* = 0.01, 0.1, 0.5, no data sets showed significant positive selection by either method. When the true *ω* = 2 and *t* = 0.5, 1, 5, both methods correctly detect positive selection in almost 100% of the replicate data sets, so that the power of detecting positive selection is high in both methods but with the LRT having more power (table 1). When *ω* = 2 and *t* = 0.1, positive selection is detected in 35% and 61% of data sets by the Bayesian and ML methods, respectively. In this case, given the short sequence distance, the prior has quite some impact on the ability of the Bayesian method to detect selection. In particular, the prior mean (*ω* = 0.5) is smaller than the true value (*ω* = 2), so that 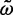 is shrunk away from 1.

### Analysis of Real Data

We applied both ML and Bayesian methods to estimate *ω* and *t* for pairwise alignments of protein-coding genes from four mammalian genomes (human, chimpanzee, mouse, and rat) and from three bacterial genomes (*E. coli* O157:H7, *E. coli* K-12, and *S. typhimurium* LT2). In all analyses, the codon frequencies were estimated by using the observed codon frequencies in the genes (the F61 model).

#### Analysis of the Mammalian Data Set

**Fig. 4.**
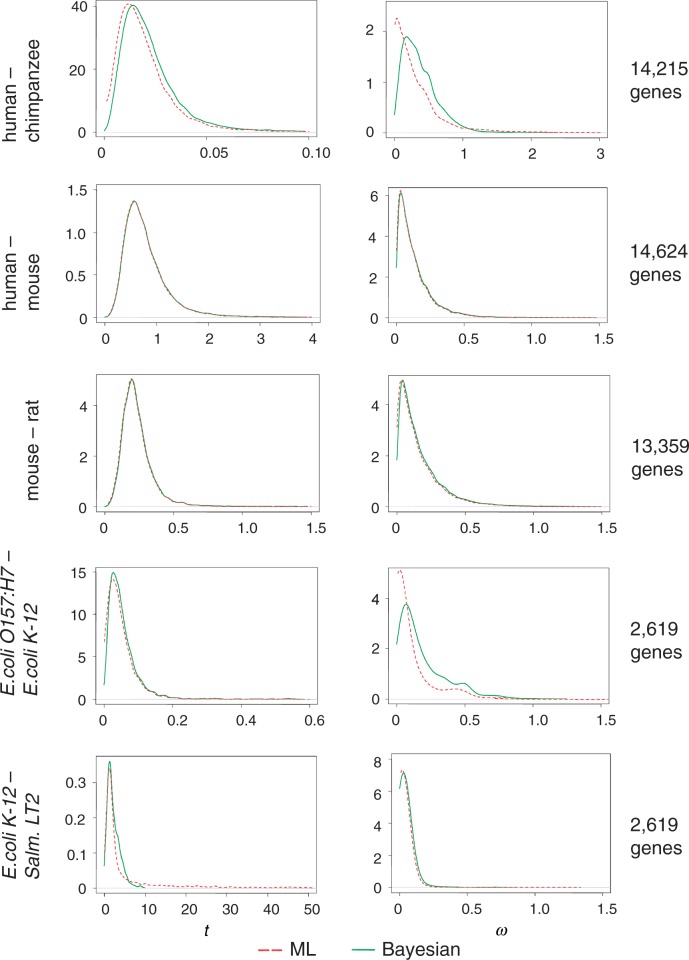
Distributions (smoothed histograms) of Bayesian and ML estimates of *t* and *ω* from mammalian and bacterial pairwise gene comparisons. Numbers of genes analyzed in each comparison are shown in the right part of the figure.

We conducted three sets of pairwise comparisons: human versus chimpanzee, human versus mouse, and mouse versus rat. Figure 4 shows the distributions (smoothed histograms) of posterior means and the MLEs of *t* and *ω* in those comparisons. In the human–chimpanzee comparison, the Bayesian *ω* estimates are slightly shifted to the right compared with the MLEs for low *ω* values and shifted to the left for high *ω* values. The mean, median, and 25% and 75% percentiles of the Bayesian estimates are 0.369, 0.320, and (0.180, 0.500) whereas those of the MLEs are 0.307, 0.193, and (0.062, 0.411) (table 3). The human and chimpanzee genes are very similar and the patterns are similar to those observed in computer simulation for low *t* values. Moreover, there are 377 and 2,507 gene alignments in which 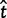 = 0 and 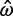 = 0, respectively, as well as 2 and 423 alignments where 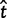 = ∞ and 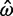 = ∞, respectively. The Bayesian method does not produce any such extreme estimates. The number of genes in which the *ω* estimate is >1 is 1,121 for ML and 299 for the Bayesian method (table 4). The discrepancy is the result of two effects, a short evolutionary distance and a short sequence length, both indicating a lack of information and greater influence from the prior. Genes with 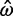 > 1 tend to be small (median sequence length 313 codons, compared with 454 codons for all genes). For example, one gene among those 1,121 with 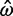 > 1 has 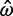 = 1.22 (95% confidence interval—CI 0.37–4.01) and posterior mean 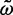 = 0.93 (95% credibility interval—CI 0.36–2.43). This gene has a length of 262 codons and has a small evolutionary distance with 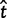 = 0.043 (95% CI 0.024–0.077) and 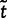 = 0.047 (95% CI 0.027–0.082), so that the prior has an impact. Another gene has 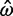 = 1.27 (95% CI 0.75–2.16) and 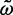 = 1.13 (95% CI 0.60–2.13). This gene is 257 codons in length and the ML and Bayesian distance estimates are 0.17 (95% CI 0.13–0.24) and 0.18 (95% CI 0.13–0.24), respectively. The second gene has a similar length to the first but because the sequence distance is greater, the prior is much less important. In a third gene, of length 1,019 codons, the MLEs are 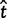 = 0.041 (95% CI 0.030–0.056) and 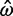 = 1.27 (95% CI 0.77–2.07), compared with the Bayesian estimates 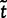 = 0.042 (95% CI 0.031–0.057) and 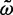 = 1.13 (95% CI 0.59–2.14). In this case, the effect of the prior is unimportant, because the gene is long.

**Table 3.** Descriptive Statistics of Bayesian (top, underlined) and ML (bottom) Estimates of *t* and 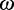 from Pairwise Comparisons of Protein-Coding Genes from Mammalian Species and Bacterial Strains.

		*ω*							*t*						
		Mean	SD	Quartiles			*N*_0_	*N*_∞_	Mean	SD	Quartiles			*N*_0_	*N*_∞_
	No. of Genes			25%	50%	75%					25%	50%	75%		
Human–chimpanzee	14,215	0.369	0.246	0.180	0.320	0.500	0	0	0.025	0.072	0.013	0.019	0.028	0	0
		0.307	0.418	0.062	0.193	0.411	2507	423	0.022	0.042	0.010	0.016	0.025	377	2
Human–mouse	14,624	0.130	0.125	0.044	0.093	0.176	0	0	0.812	0.574	0.503	0.691	0.958	0	0
		0.126	0.157	0.040	0.089	0.170	221	0	0.849	1.252	0.499	0.686	0.952	0	30
Mouse–rat	13,359	0.168	0.168	0.055	0.118	0.228	0	0	0.242	0.179	0.163	0.215	0.281	0	0
		0.159	0.180	0.046	0.108	0.215	509	0	0.238	0.232	0.161	0.212	0.278	0	3
*Escherichia coli* K-12–*E.coli* O157	2,619	0.179	0.170	0.055	0.116	0.252	0	0	0.080	0.354	0.026	0.043	0.068	0	0
		0.099	0.174	0.001	0.034	0.110	912	31	0.073	0.527	0.020	0.038	0.064	121	6
*E. coli* K-12–*Salmonella typhimurium* LT2	2,619	0.037	0.042	0.016	0.025	0.042	0	0	2.261	1.546	1.153	1.836	3.129	0	0
		0.025	0.042	0.006	0.018	0.032	164	0	5.052	8.481	1.087	1.748	4.066	0	217

Note.—The F61 model is used for codon frequencies. Results for ML have been calculated after removing the infinite estimates. *N*_0_ is the number of genes with the MLE 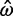 or 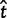 = 0, whereas *N*_∞_ is the number of genes with the MLE 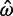 or 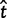 = ∞.

**Table 4.** The Numbers of Genes with 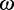 Estimate Greater or Less than 1 Using the Bayesian and ML Methods.

Data			Bayesian		
			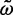 < 1	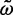 > 1	*N*_L_
Human–chimpanzee		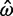 < 1	13,094	0	78
		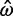 > 1	822	299	
		*N*_B_		3	
Human–mouse		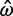 < 1	14,617	0	2
		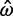 > 1	1	6	
		*N*_B_		2	
Mouse–rat	ML	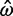 < 1	13,313	0	5
		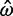 > 1	10	36	
		*N*_B_		2	
*Escherichia coli* K-12–*E. Coli* O157		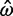 < 1	2,574	0	0
		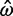 > 1	43	2	
		*N*_B_		0	
*E. coli* K-12–*Salmonella typhimurium* LT2		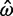 < 1	2,617	0	0
		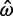 > 1	2	0	
		*N*_B_		0	

Note.—*N*_L_ is the number of genes with statistically significant 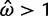 based on the LRT at the 5% level (one-sided with critical value 2.71) in the likelihood method, whereas *N*_B_ is the number of genes with *P*(*ω* > 1 | *x*) > 0.95 in the Bayesian analysis.

Among the 1,121 genes with 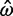 > 1 only 78 have statistically significantly evidence of positive selection, based on the LRT (*α* = 5%) (table 4). All the 78 genes have the posterior mean 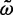 > 1. Moreover, out of them, three showed strong evidence of positive selection in the Bayesian analysis, with *P*(*ω* > 1 | *x*) > 0.95 (table 4). The difference (78 vs. 3 genes) in the number of genes with *ω* > 1 between the ML and the Bayesian method is consistent with the general expectation that the LRT tends to reject the null more readily than the Bayesian analysis. It is also consistent with the results observed in the computer simulations for *t* = 0.1 and *ω* = 2. We note that the three genes significant in the Bayesian analysis have fairly large sequence divergences, with 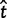 ≈ 0.1, whereas the other 75 genes (for which the LRT is significant but the Bayesian evidence is not strong) have highly similar sequences, with 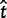 < 0.07 (with median 0.021).

In the human–mouse comparison, the ML and Bayesian estimates are very similar. The sequence divergence is intermediate, the data are informative, and the prior does not have a noticeable impact. There are very few cases where the MLEs are extreme (0 or ∞). Also, the number of genes showing *ω* estimates >1 are nearly the same between the two methods (7 vs. 6) and the same two genes show significant evidence for positive selection by both methods. The mouse–rat comparison shows similar patterns to the human–mouse comparison: in both cases, the sequences are moderately divergent and the data are informative.

To examine the sensitivity of posterior estimates of *t* and *ω* to the prior, we reanalyzed the human–chimpanzee and human–mouse alignments using two alternative priors: AP1 and AP2. The first alternative prior (AP1) is *t* ∼ *G*(2, 2) and *ω* ∼ *G*(2, 4). This has the same means as the default prior of equation (2) but the prior is more informative because of the larger shape parameter (2 vs. 1.1). In the second alternative prior (AP2), we used 2 for the shape parameter, but chose the rate parameter such that the prior mean roughly matches the median of the MLEs for all genes (table 3). Thus, for the human–chimpanzee comparison, AP2 is *t* ∼ *G*(2, 100), with the prior mean 0.02 (while the median of MLEs of *t* is 0.016), and *ω* ∼ *G*(2, 10), with the prior mean 0.2 (while the median of MLEs of *ω* is 0.193). For the human–mouse comparison, AP2 specifies *t* ∼ *G*(2, 3), with the prior mean 0.67 (while the median of the MLEs is 0.686) and *ω* ∼ *G*(2, 20), with the prior mean 0.1 (the median of the MLEs is 0.089). While it is in general not advisable to use the data to specify the prior, we note that in specific comparisons, some prior information may be available. For example, between the human and the chimpanzee, the distance *t* is very likely to be smaller than 0.1.

**Fig. 5.**
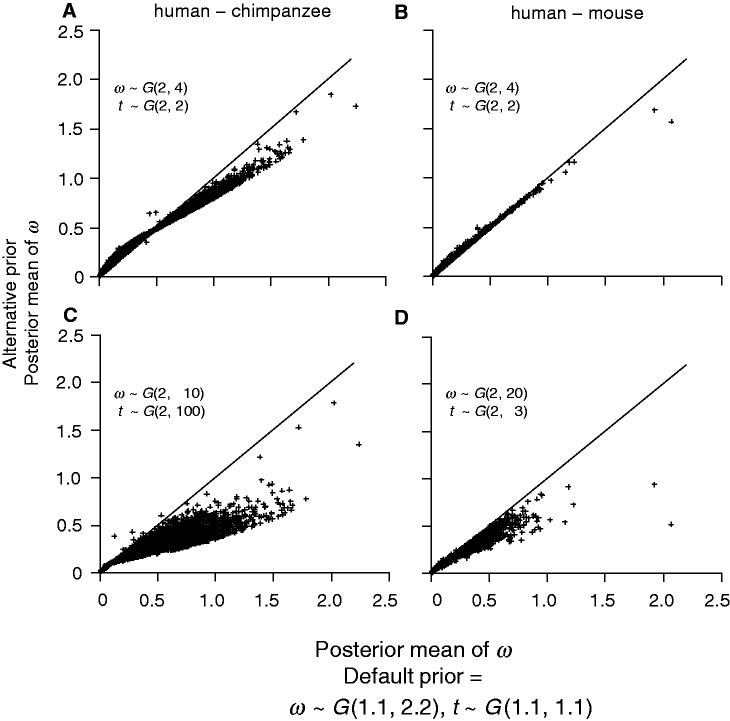
Bayesian estimates of *ω* for the human–chimpanzee (A and C) and human–mouse (B and D) comparisons using two alternative priors plotted against estimates using the default prior (eq. 2). The alternative priors are: (A and B) *ω* ∼ *G*(2, 4), *t* ∼ *G*(2, 2); (C) *ω* ∼ *G*(2, 10), *t* ∼ *G*(2, 100); and (D) *ω* ∼ G(2, 20), *t* ∼ G(2, 3).

**Fig. 6.**
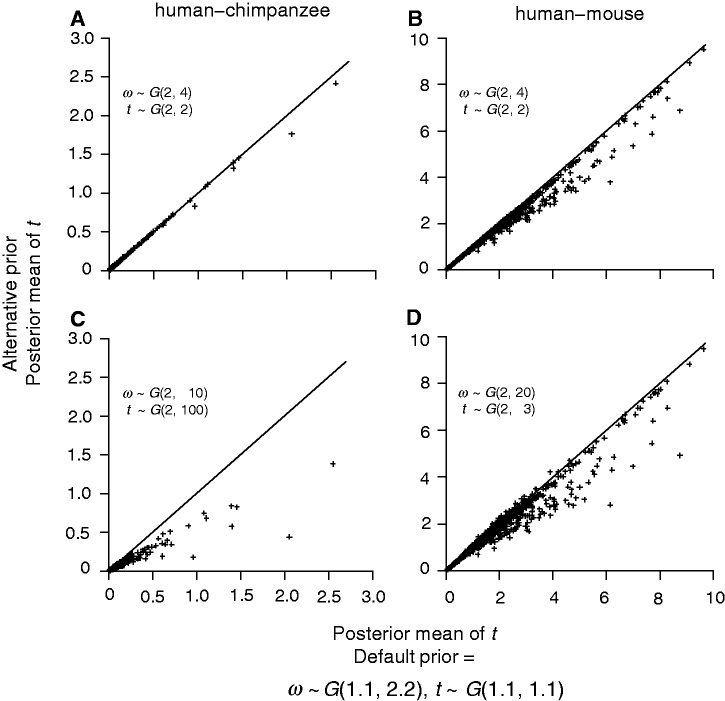
Bayesian estimates of *t* for the human–chimpanzee (A and C) and human–mouse (B and D) comparisons using different gamma priors. The alternative priors are as in figure 5.

Posterior estimates of *ω* and *t* from the analysis using the default and alternative priors are illustrated in figures 5 and 6. In the human–chimpanzee comparison, the impact of the prior is apparent. The Bayesian *ω* estimates using the AP1 are higher than those using the default prior for low *ω* values (*ω* < 0.5) and lower for high *ω* values (*ω* > 0.5) (fig. 5*A*). With a more informative prior (shape parameter 2), the posterior means are closer to the prior mean 0.5. For the human–mouse comparison estimates under AP1 are close to those under the default prior (fig. 5*B*). The Bayesian estimates of *t* are less affected by the change in the prior in both comparisons and the estimates are approximately the same for the majority of the genes (fig. 6*A* and *B*). Prior AP2 has a more significant effect. In both comparisons, the Bayesian estimates of *ω* are smaller than those obtained using the default prior for almost all genes (fig. 5*C* and *D*). The priors are more informative (with shape parameter *α* = 2) and have lower means (0.2 and 0.1 for the human–chimpanzee and human–mouse comparisons, respectively, instead of 0.5) and thus affect posterior estimates more than the default prior. The effect is more apparent in the human–chimpanzee comparison because of the smaller sequence distances. Posterior estimates of *t* are less affected by the change in the prior (fig. 6*C* and *D*). In summary, the prior affects posterior estimates of *ω* when the genes are not informative about *ω* and does not affect significantly the posterior estimates of *t*.

#### Analysis of the Bacterial Data Set

We conduct two pairwise comparisons: *E. coli* K-12 versus *E. coli* O157:H7 and *E. coli* K-12 versus *S. typhimurium* LT2. Note that the two strains of *E. coli* have the same evolutionary distance from the *Salmonella*.

The sequences from the two *E. coli* strains are very similar, and the prior has an impact on Bayesian estimates, similar to the comparison of the human and chimpanzee genes. The mean, median, and 25% and 75% percentiles of the Bayesian *ω* estimates are 0.179, 0.116, and (0.055, 0.252) while the corresponding results for the MLEs are 0.099, 0.034, and (0.001, 0.110). The two methods are thus very different in analysis of those genes. Also, the MLE 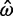 = 0 in 912 genes and 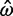 = ∞ in 31 genes. None of the genes with 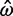 > 1 is statistically significant at the *α* = 5% significance level according to the LRT and none has *P*(*ω* > 1 | *x*) > 0.95 (table 4). The gene sequences from the *E. coli* K-12 and *Salmonella* are quite divergent. In most genes, the two methods produced similar estimates (fig. 4). However, some genes are very divergent with the MLE 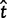 = ∞ in 217 genes.

## Discussion

We suggest that if possible one should conduct joint comparative analysis of multiple protein-coding gene sequences on a phylogeny, instead of pairwise comparisons. In particular, a number of LRTs have been developed to detect positive selection that affects particular evolutionary lineages on the phylogeny or individual sites in the protein (see, e.g., Yang [2006a] and Cannarozzi and Schneider [2012], for reviews). To apply such tests of positive selection, it is essential to use multiple sequences, as a pair of sequences hardly contains enough information for the tests to have any power (e.g., Yang 2006b). Some proteins may evolve in an episodic manner and thus adaptive episodes may not be detected in pairwise comparisons, especially when the sequences are distantly related (Messier and Stewart 1997). In a pairwise comparison, positive selection is detected only if the *ω* averaged over all sites in the protein and over the whole evolutionary history connecting the two sequences is >1. This seems to be an extremely stringent criterion. Analysis of multiple sequences on a phylogeny allows one to detect episodic positive selection that affects a particular branch (Yang 1998).

Nevertheless, we note that pairwise sequence comparisons are widely used, especially in comparative genomics, sometimes to provide summary statistics of the data and sometimes because of lack of a third genome. The ML method has been used to estimate *ω* and *t* in pairwise comparisons of genes (e.g., Nielsen et al. 2005; Ge et al. 2008; Walters and Harrison 2010; Buschiazzo et al. 2012; Gladieux et al. 2013; Wang and Chen 2013). Counting methods are also used due to their simplicity (Garcia-Gil et al. 2003; Schenekar et al. 2011; Graves et al. 2013), even though they were found not to perform as well as ML in computer simulations (Yang and Nielsen 2000). Both counting and ML methods sometimes return 0 or ∞ as estimates, so that neither the expectation nor the variance of the estimates is finite. The infinity estimates of *ω* appear to be particularly confusing to many users of the methods. To avoid such extreme estimates, some authors (e.g., Novaes et al. 2008; Bajgain et al. 2011; Pellino et al. 2013) added a small arbitrary number (pseudocounts) to the numbers of synonymous and nonsynonymous substitutions before calculating *ω*. Other authors excluded genes with *d*_S_ = 0 from their analysis (e.g., Wang and Chen 2013). The Bayesian method implemented here may provide a better procedure than such ad hoc treatments. It always returns finite estimates of *ω* and *t* as the prior penalizes extreme values. Our computer simulation suggests that the Bayesian estimates of *ω* have nice statistical properties, with similar or smaller MSEs compared with the MLEs. The posterior means are close to the MLEs when the data are informative, that is, when the sequences are long and the sequence divergence is intermediate, but the differences can be large when the sequences are short and are either too similar or too divergent. Nearly identical sequences contain little information while extremely divergent sequences contain too much noise concerning *ω*. In both cases, the data are not informative and the prior has an impact on posterior estimates of *ω*. However, as sequence length increases the effect of the prior decreases irrespective of the true values of *ω* and *t*. Our Bayesian method is used for the analysis of only two sequences. A Bayesian method for the analysis of multiple sequences in a phylogeny requires calculation of high-dimensional integrals and is not pursued here.

We emphasize that MLEs 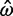 = ∞ should not be taken as evidence for positive selection (*ω* > 1) because the extreme estimate may well be due to chance effects when the numbers of changes are small. Instead, positive selection can be claimed only if the LRT is significant in the ML framework or when *P*(*ω* > 1 | *x*) > 0.95 in the Bayesian analysis.

### Program Availability

The Bayesian method of this article is implemented in the CODEML program in the PAML package. The program allows the user to specify gamma priors for *t* and *ω*. Although the Bayesian method is computationally more intensive than ML, it remains fast enough for large-scale screening. It takes 1–2 s to analyze one pair of sequences on a modern PC.

## Methods and Materials

### Theory

We use a simplified version of the model of Goldman and Yang (1994) to model the evolution of codon sequences (Yang and Nielsen 1998). The model accounts for the genetic code structure, the transition/transversion rate ratio, the codon frequencies as well as the *d*_N_/*d*_S_ rate ratio *ω*. The instantaneous substitution rate from codon *i* to codon *j* (*i* ≠ *j*) is given by
(5)
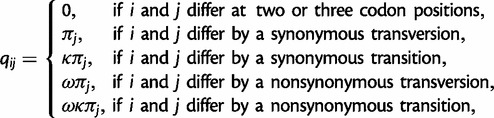

where *π_j_* is the equilibrium frequency of codon *j*. Stop codons are not considered (they are assumed not to occur within protein-coding genes). Therefore, the substitution rate matrix *Q = *{*q_ij_*} is of size 61 × 61 for the standard genetic code. The rate matrix is scaled so that the average rate of codon substitution equals 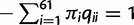, and thus time is measured by the expected number of nucleotide substitutions per codon site. We use standard theory to calculate the transition probability matrix over time *t* as *P*(*t*)* = *exp(*Qt*). The likelihood function on a pairwise sequence alignment *x* is
(6)
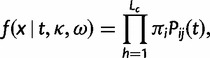

where *i* and *j* are the observed codons in the two sequences at site *h* and *L*_c_ is the number of codons.

The joint posterior distribution of *ω* and *t* is given by equation (1). If *κ* is a parameter in the model we replace it with its MLE (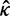). If the two sequences are identical so that 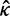 is not unique, we fix it at 2. Besides the posterior means of *ω* and *t* given in equations (3) and (4), we also calculate the posterior variances and covariance
(7)
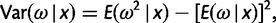

(8)
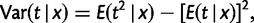

(9)
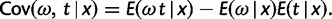



Thus, six double integrals need to be computed, one for the normalizing constant C, and five for the different expectations in equations (3), (4), and (7)–(9).

Consider the calculation of the normalizing constant C. All other integrals are calculated in the same way. We write *g*(*t*, *ω*) = *f*(*x*|*t*,* ω*) *f*(*t*,* ω*). To avoid overflows and underflows, we set *h*(*t*, *ω*) = exp{log[*g*(*t*, *ω*)]−*l*_max_}, where *l*_max_ is the maximum of *g*(*t*, *ω*), a constant chosen for scaling. The normalizing constant can then be written as
(10)
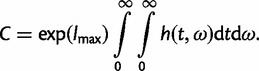



We use the Gaussian quadrature method to calculate all integrals numerically, which uses Legendre polynomials to approximate any continuous integrand function *f*(*x*, *y*):
(11)
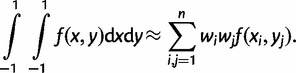



The weights *w_i_* and *w_j_* and the points *x_i_* and *y_j_* at which the integrand is evaluated are predetermined given the total number of points *n*. In our case, the limits of the integrals are 0 and ∞ and we have to use a transformation to map the (0, ∞) limits to (−1, 1). A much more serious problem is that the integrand *g* may be spiky (i.e., it is highly concentrated in a very small interval) and the approximation will be very poor if the sampled points miss the spike in the integrand. The rationale behind our transformation is to find a probability density function (PDF) that has a similar shape to the integrand *g*(*t*, *ω*) and then we use its cumulative distribution function (CDF) to transform the integrand. Note that if the chosen PDF matches the posterior exactly, the new integrand will become perfectly flat after the transformation. The logistic distribution is used for that purpose.

Let *x*_1_ = log*t* ∼ Logistic(*μ*_1_, *σ*_1_) and *x*_2_ = log*ω* ∼ Logistic (*μ*_2_, *σ*_2_). For any random variable *x* ∼ Logistic(*μ*, *σ*) the CDF is 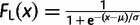. Thus, for equation (10), we use the following transformation (change of variables):
(12)
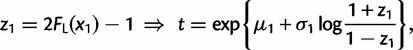

(13)
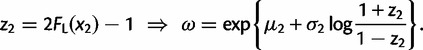

Thus, equation (10) becomes
(14)
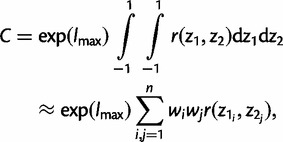

where 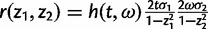 and *t* and *ω* are given by equations (12) and (13), respectively. We transform all other integrals in equations (3), (4), and (7)–(9) in the same way. Thus, we have
(15)
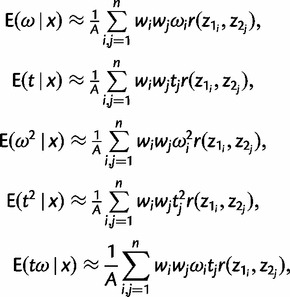

where *A* = *C*exp(−*l*_max_). Notice that the exponential term exp(*l*_max_) cancels out during calculations.

Our Bayesian calculation is performed after the MLEs are obtained. Thus if both 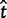 and 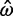 are finite, away from 0 and the observed *p*_S_ and *p*_N_ (proportion of synonymous differences per synonymous site and proportion of nonsynonymous differences per nonsynonymous site, respectively) are <0.74, we set *μ*_1_ = log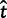, *μ*_2_ = log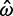, 
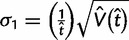
, and 
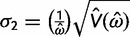
. The variances V(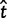) and V(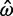) are estimated using the Nei and Gojobori (1986) method. Because the Nei and Gojobori method uses the Jukes and Cantor (1969) nucleotide substitution model (JC69) to correct for multiple hits, the use of 0.74 as an upper limit for the *p*_S_ and *p*_N_ guarantees an adequate estimation of V(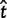) and V(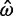).

In all other cases, we find numerically the point (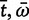) that maximizes log{*g*(*t*, *ω*)}. We calculate the Hessian matrix at this point using the second-order difference method and use the inverse of the Hessian to estimate the variances V(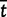) and V(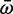). Then, we set *μ*_1_ = log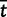, *μ*_2_ = log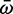, 
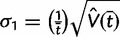
, and 
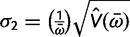
. Notice that because of our choice of the prior, log(*g*) always has a mode and thus the optimization algorithm returns a point away from (0, 0).

We use the same number of points *n* for both parameters *ω* and *t* in the Gaussian quadrature. With *n* = 32, each sum in equation (15) requires 32 × 32 = 1,024 evaluations of the *r*(*z*_1_, *z*_2_) function. Tests suggest that using 32 points achieves high accuracy. The use of more points increases the computational time radically since evaluation of *r*(*z*_1_, *z*_2_) requires evaluation of the likelihood which is computationally expensive. Moreover, we use the same techniques described above to calculate the posterior probability *P*(*ω* > 1 | *x*) = 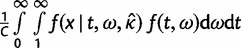, as a Bayesian equivalent of the LRT for positive selection indicated by *ω* > 1.

### Real Data Analysis

Both the new Bayesian method of this article and the ML method of Goldman and Yang (1994) were applied to compare protein-coding genes from mammalian species and bacterial strains. The mammalian data set is a subset of the data analyzed by dos Reis et al. (2012). There are 14,218 genes from the human and chimpanzee, with the sequence length ranging from 39 to 8,797 codons; 14,631 genes from the human and mouse with the sequence length from 13 to 8,787 codons; and 13,371 genes from the mouse and rat with the sequence length from 14 to 7,798 codons. The protein-coding sequences from the genomes of *E. coli* O157:H7, *E. coli* K-12, and *S. typhimurium* LT2 were downloaded from GenBank (accession numbers: U_00096, NC_002655, and NC_003197). Orthologous genes among the three genomes were identified by using the program BLAT (Kent 2002) to extract the best reciprocal hits. Only orthologs present in all three genomes are used. This bacterial data set consists of 2,631 genes from each strain, with the sequence length ranging from 20 to 1,485 codons. Codons involving alignment gaps and ambiguity nucleotides were removed prior to analyses. Moreover, genes with sequence length of 50 codons or less were excluded from the analysis. The number of genes analyzed in each comparison is reported in table 3 and figure 4.
